# Proteogenomic mapping of *Mycoplasma hyopneumoniae* virulent strain 232

**DOI:** 10.1186/1471-2164-15-576

**Published:** 2014-07-08

**Authors:** Ken Pendarvis, Matthew P Padula, Jessica L Tacchi, Andrew C Petersen, Steven P Djordjevic, Shane C Burgess, F Chris Minion

**Affiliations:** Department of Veterinary Microbiology and Preventive Medicine, Iowa State University, Ames, IA USA; School of Animal and Comparative Biomedical Sciences, University of Arizona, Tucson, AZ USA; ithree institute, University of Technology, Sydney, Australia; Proteomics Core Facility, Faculty of Science, University of Technology, Sydney, Australia; Bio5 Institute, School of Animal and Comparative Biomedical Sciences, University of Arizona, Tucson, AZ USA

**Keywords:** *Mycoplasma hyopneumoniae*, Proteome, Swine pathogen, Proteogenomic, Mapping, Mass spectrometry

## Abstract

**Background:**

*Mycoplasma hyopneumoniae* causes respiratory disease in swine and contributes to the porcine respiratory disease complex, a major disease problem in the swine industry. The *M. hyopneumoniae* strain 232 genome is one of the smallest and best annotated microbial genomes, containing only 728 annotated genes and 691 known proteins. Standard protein databases for mass spectrometry only allow for the identification of known and predicted proteins, which if incorrect can limit our understanding of the biological processes at work. Proteogenomic mapping is a methodology which allows the entire 6-frame genome translation of an organism to be used as a mass spectrometry database to help identify unknown proteins as well as correct and confirm existing annotations. This methodology will be employed to perform an in-depth analysis of the *M. hyopneumoniae* proteome.

**Results:**

Proteomic analysis indicates 483 of 691 (70%) known *M. hyopneumoniae* strain 232 proteins are expressed under the culture conditions given in this study. Furthermore, 171 of 328 (52%) hypothetical proteins have been confirmed. Proteogenomic mapping resulted in the identification of previously unannotated genes *gatC* and *rpmF* and 5-prime extensions to genes mhp063, mhp073, and mhp451, all conserved and annotated in other *M. hyopneumoniae* strains and *Mycoplasma* species. Gene prediction with Prodigal, a prokaryotic gene predicting program, completely supports the new genomic coordinates calculated using proteogenomic mapping.

**Conclusions:**

Proteogenomic mapping showed that the protein coding genes of the *M. hyopneumoniae* strain 232 identified in this study are well annotated. Only 1.8% of mapped peptides did not correspond to genes defined by the current genome annotation. This study also illustrates how proteogenomic mapping can be an important tool to help confirm, correct and append known gene models when using a genome sequence as search space for peptide mass spectra. Using a gene prediction program which scans for a wide variety of promoters can help ensure genes are accurately predicted or not missed completely. Furthermore, protein extraction using differential detergent fractionation effectively increases the number of membrane and cytoplasmic proteins identifiable my mass spectrometry.

**Electronic supplementary material:**

The online version of this article (doi:10.1186/1471-2164-15-576) contains supplementary material, which is available to authorized users.

## Background

*Mycoplasma hyopneumoniae* is the etiological agent of porcine enzootic pneumonia [1], causing substantial economic losses to the pig industry through reduced average daily weight gain and efficiency of feed utilization, prophylactic and therapeutic costs, and mortality [1, 2]. When co-infections occur with a secondary (bacterial or viral) infection, the respiratory disease is more severe and has been designated as porcine respiratory disease complex [1], an even more devastating disease. The virulence factors of *M. hyopneumoniae* are largely unknown and to better understand the mechanisms involved, we are studying genetic processes in *M. hyopneumoniae* both *in vitro* and *in vivo* [3–8]. Recent microarray studies of global transcriptional changes clearly show that under the culture conditions used in this study, 627 of the 691 known protein coding genes are transcribed [4–8]. *M. hyopneumoniae* also responds to environmental changes, and under various stressors, all annotated genes are transcribed [4–8]. Further, a recent study from our laboratory also shows that intergenic regions are transcribed [9]. The genome for *M. hyopneumoniae* has been sequenced [10–12], and from that sequence, 691 protein coding genes have been annotated in strain 232. Our next steps in completing the picture of gene expression in *M. hyopneumoniae* has been to construct a proteogenomic map of *M. hyopneumoniae* and to survey its metabolic capabilities. This will assist in annotating the genome and identifying any potential genes missed in the original annotation that could explain the extent of intergenic transcription observed by Gardner *et al*. [9]. To this end we have employed both one and two dimensional liquid chromatography nanospray ionization tandem mass spectrometry (1D and 2D-LC NSI MS/MS).

## Results

### Identified proteins

Protein samples were analyzed using two mass spectrometers, an LTQ Velos Pro (Velos) and an LTQ FT Ultra (FT). Samples were run on the FT as part of a post translational modification study beyond the scope of this manuscript but are included here for protein identification purposes only. X!tandem [13] and OMSSA [14] peptide identifications from the Velos and FT were combined, resulting in 8,607 peptide sequences identified from 46,166 peptide-spectrum matches with a maximum false discovery rate of 0.53%. Subsequently, 483 proteins (70%) of the currently annotated 691 protein coding genes in *M. hyopneumoniae* strain 232 were identified; 171 of 328 (52%) hypothetical proteins have been confirmed. Supporting Information Additional file 1: Table S1 shows all protein coding genes in the original order of the genome annotation with those identified in this study marked verified. Protein coverage and the number of unique peptide sequences identifying each verified protein are included. Detailed peptide and protein identifications with confidence scores are provided in Supplemental Information Additional file 2: Proteome search results.

### Differential detergent fractionation

Differential detergent fractionation (DDF) was used to sequentially extract proteins based on hydrophobicity. A gene ontology (GO) enrichment was performed on proteins identified from the different DDF fractions, as well as those from the non-DDF, FT runs. Table 1 shows the number of proteins matching several important GO cellular component categories for 1) all annotated proteins, 2) each DDF fraction, 3) all fractions and 4) the non-DDF runs. DDF and non-DDF methods resulted in no difference in number of intracellular, chromosomal and ribosomal protein identifications. However, DDF provided a 29% increase in the number of membrane proteins and 12% increase in cytoplasmic proteins. Furthermore, the sodium dodecyl sulphate (SDS) and insoluble fractions contained 80% more membrane proteins than digitonin and Tween 20. A similar but less pronounced trend was also seen in cytoplasmic proteins.

**Table 1:** **GO term protein counts organized by DDF fraction**

GO term	Annotated proteome	Non DDF	All fractions	Digitonin	Tween	SDS	Insoluble
**Membrane**	**61**	**34**	**44**	**20**	**24**	**43**	**36**
Integral component of membrane	48	15	29	15	14	28	23
**Cytoplasm**	**97**	**78**	**87**	**74**	**54**	**79**	**81**
**Intracellular**	**45**	**39**	**39**	**35**	**32**	**33**	**35**
**Ribosome**	**47**	**43**	**43**	**42**	**37**	**38**	**40**
Large ribosomal subunit	5	5	5	5	5	5	5
Small ribosomal subunit	7	7	7	7	7	7	7
**Chromosome**	**4**	**4**	**4**	**3**	**3**	**4**	**4**

### Proteogenomic mapping

To complement the identification of known and predicted proteins in *M. hyopneumoniae* strain 232, and subsequently identify possible unannotated open reading frames (ORFs) and errors in the current annotations, mass spectra were searched using X!tandem and OMSSA against a 6-frame genomic translation. The genomic searches resulted in 7,765 peptide sequences from 42,330 matched spectra with a maximum false discovery rate of 0.73%. After combining both the protein and genome search results, 9,039 unique peptide sequences were identified from 47,674 positively matched spectra across all eight samples. Detailed peptide identifications with confidence scores are provided in Supplemental Information Additional file 3: Genome search results.

Peptide sequences were mapped to the *M. hyopneumoniae* strain 232 genome and categorized by location (Table 2). Proteogenomic mapping revealed two areas of intergenic translation, annotated in other stains of *Mycoplasma hyopneumoniae* as genes *gatC* and *rpmF*. Five-prime extensions to annotated genes were identified in mhp063, mhp073, and mhp451; BLAST results indicate these extensions are present in genes in other strains. The Prodigal prokaryotic gene predicting software also predicted the previously unannotated genes and extensions in agreement with proteogenomic mapping (Table 3).

**Table 2:** **Gene model alterations and novel mappings**

5' extensions						
**Locus**	**Old Start**	**New Start**	**Stop**	**Direction**	**Peptides**	**Notes**
mhp063	74727	74166	76196	+	4	Extension present in strains 7448, 7422, 168 and J
mhp073	91551	91023	92147	+	4	Extension present in strains 7448 and J
mhp451	555365	555692	554796	-	3	Extension present in strains 7448, 7422 and J
**Intergenic mappings**						
**Start**	**Stop**	**Direction**	**Peptides**	**Notes**		
34617	34910	+	4	Unannotated gene; overlaps mhp029; Blast indicates gene is *gatC* in other strains		
120237	120040	-	3	Unannotated gene; Blast indicates gene is *rpmF* in strain J

**Table 3:** **Comparison of Prodigal and proteomgenomic mapping coordinates**

5' extensions					
**Locus**	**PGM Start**	**Prodigal Start**	**Stop**	**Direction**	RBS Motif*
mhp063	74166	74166	76196	+	AGxAGG/AGGxGG
mhp073	91023	91023	92147	+	GGA/GAG/AGG
mhp451	555692	555692	554796	-	AGGAG
**Intergenic mappings**					
**Gene**	**PGM Start**	**Prodigal Start**	**Stop**	**Direction**	RBS Motif*
*gatC*	34617	34617	34910	+	GGxGG
*rpmF*	120237	120237	120040	-	AGGA

*An "x" in the motif indicates a mismatch is allowed.

## Discussion

### Identified proteins

One other group has performed a recent global proteomics analysis of *M. hyopneumoniae* similar to our study; Pinto *et al*. reported identifying 35% of the proteins in strains J, 7422 and 7448 [15, 16]. Jaffe *et al*. identified 81% of the proteins of the related species *M. pneumoniae* [17] and Yuan *et al.* identified 51% of the proteins in *M. suis* [18]. By combining all of our samples, we identified 70% of the proteins in *M. hyopneumoniae* strain 232. The increase in proteome coverage from 35% to 70% achieved by our study is likely due to the large number of replicates (eight total) compared to a maximum of three stated in the other studies, and the dual instrument, dual sample preparation approach used in our analysis.

Many of the proteins identified in this study are only computationally predicted and, as such, given the "hypothetical" annotation. Our high throughput experimental annotation confirms that 171 (52%) of these genes are translated. From our previous transcriptome studies [4–8], evidence shows that 627 of the 691 protein coding genes are transcribed under the growing conditions in this study. We failed to identify 208 of the 691 total protein coding genes because they: 1) are expressed at very low levels or not at all under the culture conditions; 2) were not included as they did not make our stringent identification criteria; 3) were mis-annotated or contained sequencing errors; 4) contain peptides which are inherently resistant to electrospray ionization; or 5) in the case of the hypothetical product of mhp383, produce no tryptic peptides of suitable size (greater than 6 and less than ~40 amino acids) identifiable using the analysis techniques employed in this study.

### Differential detergent fractionation

*M. hyopneumoniae* cells were subjected to DDF and non-DDF sample preparation techniques in this study. Membrane proteins are frequently insoluble in most detergent solutions used in sample preparation [19], and it is reasonable to expect an abundance of membrane proteins in the SDS fractions and insoluble cellular debris. DDF was performed using 3 detergents of increasing strength: digitonin, Tween 20 and SDS. Table 1 shows the number of annotated proteins in several major GO categories, as well as numbers identified in DDF and non-DDF methods. There are 61 annotated membrane related proteins in *M .hyopyneumoniae* strain 232. Thirty four of these proteins (56%), were identified in the non-DDF analysis, verses 44 (72%) from DDF, amounting to a 30% increase. Furthermore, evidence shows 80% more membrane proteins being identified in the SDS and insoluble fractions. A similar but less pronounced trend was seen with cytoplasmic proteins, with DDF providing a 12% increase in protein identifications over non-DDF. A 25% increase in cytoplasmic proteins was seen in the SDS and insoluble fractions over digitonin and Tween 20. Protein concentration in the digitonin fractions was about 10-fold greater than that the other fractions, indicating that less abundant, hydrophobic membrane and cytoplasmic proteins could be masked from detection in a non-DDF method. All fractions were normalized to 20 μg before digestion, therefore enriching the analysis with membrane proteins by over representing Tween 20, SDS and insoluble proteins. It is interesting that more cytoplasmic proteins were identified in the more hydrophobic fractions. Since cytoplasm is composed of cytosol, ogranelles and various other inclusions, it is reasonable to expect proteins from the more organized structures to be less soluble that those in the cytosol. This easily explains the increase in numbers with hydrophobicity. No other GO categories showed such an increase in protein identifications with DDF. This evidence shows that samples can be enriched with membrane proteins by using a series of detergents to solublize proteins based on increasing hydrophobicity, and subsequently normalizing on protein quantity. Much future work is required to provide a more detailed GO analysis of M. hyopeumoniae proteins, as only 229 (31%) of known proteins have cellular component annotation, most of which are very generalized. Better annotation would be helpful in categorizing which cellular components are easily separated based on DDF methods.

### Proteogenomic mapping

Proteogenomic mapping indicates that the current *M. hyopneumoniae* strain 232 genome is well annotated with only 1.8% of peptide mappings not belonging to currently known genes. The identification of two unannotated genes, *gatC* and *rpmF*, is surprising considering the small genome size and high degree of genetic similarity between *M. hyopneumoniae* strains. The 5-prime extensions, present in annotated genes from other strains, were not predicted in strain 232 likely due to bias in the ORF finding algorithm used. ORF finders typically scan the 6 frames of a genomic sequence and predict ORFs based on distance between start and stop codons. Prodigal has an advantage over this type of ORF finder in that it scans for ribosomal binding sites (RBS). As shown in Table 3, each gene predicted by Prodigal, has a different RBS motif. All of these motifs are present in other proteins correctly predicted in the original annotations. If the original prediction did not rely on RBS detection, failure to determine the true start codons for certain ORFs would be more likely, explaining the 5' extensions detected by proteogenomic mapping. As for the unannotated genes, *rpmF* and *gatC*, it is unclear as to why these were missed. They are rather short genes, r*pmF* and *gatC* being 197 and 293 bases in length respectively, but 23 annotated genes are shorter than both. *gatC* overlaps the 5' end of *gatA*, but 172 other annotated genes overlap another. These difficult to explain instances are good reasons to validate predictions with proteomic and transcriptomic data.

Our previous study aimed at detecting intergenic transcription in *M. hyopneumoniae* found evidence for 321 instances of intergenic transcription [9]. We have evidence of transcription in intergenic regions upstream from mhp073 and mhp451, which supports the 5-prime extensions of these genes detected in this study. No transcription evidence was found for the 5-prime extension of mhp063. Both unannotated genes identified in this study, *gatC* and *rpmF*, also have corresponding areas of intergenic transcription. In future studies, next generation transcriptome sequencing would be a good choice to complement proteogenomic mapping and help confirm the existence of unannotated and modified genes. Unlike proteomics, transcriptomics allows gene boundaries to be clearly determined and errors in the genomic sequence to be considered when mapping reads.

Trypsin has been the enzyme of choice in proteomic analyses for many years because of is high specificity, but protein primary structures rich in lysine (K) and arginine (R) residues can result in peptides too small (<6 amino acids) to uniquely identify most proteins. Conversely, areas poor in K and R produce peptides too large (>40 amino acids) to be accurately identified by low resolution mass spectrometers, such as the LTQ Velos Pro used in this study. Secondary and tertiary protein structures resistant to denaturation can contain areas inaccessible to trypsin. Alternate protein fragmentation methods can increase protein coverage, which is beneficial in proteogenomic mapping studies which rely on maximizing coverage. Using multiple proteases which target different residues, such as trypsin, elastase and thermolysin, can result in overlapping peptides averaging 10 amino acids in length [20]. Proteinase K digestion carried out at high pH produces peptides of 6 to 20 amino acids in length, ideal for MS/MS analysis [21]. A multiple protease approach increases the likelihood accessing structurally inaccessible cleavage sites and reduces the impact of protein regions rich or poor in residues targeted by a single enzyme. A followup study employing this approach would be beneficial by potentially increasing protein coverage and further confirming unannotated areas of protein expression.

## Conclusions

Our study has provided one of the deepest proteome analyses of *M. hyopneumoniae* to date. Seventy percent of strain 232 proteins were identified and 52% of hypothetical proteins have been confirmed. Previously unannotated genes *gatC* and *rpmF* have been identified for the first time strain 232. Five-prime extensions of genes mhp063, mhp073 and mhp451 were also detected. These additions and modifications to the current annotations are conserved in other strains of *M. hyopneumoniae* and all but one, mhp063, have evidence of transcription as determined by our previous studies [4–8]. These findings illustrate how even the smallest annotated genomes are far from perfect, and future work, both transcriptomic and proteomic, is required to better understand the *M. hyopneumoniae* genome. Additionally, using a gene prediction program which detects ribosomal binding sites ensures genes are less likely to be incorrectly defined or missed during analysis. Furthermore, the use of DDF effectively enriches samples with membrane proteins by allowing proteins to be separated based on increasing hydrophobicity. Highly soluble, highly abundant proteins are concentrated in a relatively weak detergent while less soluble, less abundant membrane proteins are extracted in progressively stronger detergents. Normalizing fractions by quantity prior to trypsin digestion allows low abundance, hydrophobic proteins a greater chance of being identified. The current GO annotations for *Mycoplasma hyopneumoniae* are lacking depth and completion; much work is required to annotate the proteome both physically and functionally. Better GO annotation would provide a more thorough breakdown of protein and cellular component affinity to DDF fraction.

## Methods

### Sample preparation

*Mycoplasma hyopneumoniae* strain 232 was originally isolated from a pig infected with strain 11 [22], is fully virulent in low passage, and has been commonly used in challenge and pathogen studies in the United States. Four independent cultures (biological replicates) were grown in Friis broth [23], each split into two flasks (technical replicates), until the media color change indicated mid to late log phase of growth had been achieved (pH ~ 6.5). The cells were then centrifuged at 10,000 × *g* for 30 min, resuspended in phosphate buffered saline, and centrifuged again. This was repeated three additional times to remove medium contaminants. Of the eight replicates, six were reserved for shotgun proteomics analysis using an LTQ Velos Pro (Thermo Scientific) low resolution, high-throughput mass spectrometer, and the remaining two replicates were analyzed using an LTQ FT Ultra (Thermo Scientific) high resolution mass spectrometer.

No vertebrates subjects were involved in the culture and sample preparation of the *M. hyopneumoniae* during the course of this study. All procedures were performed within the research guidelines of the University of Arizona, Iowa State University, and the University of Technology, Sydney and did not require approval of an ethics committee.

### Low resolution mass spectrometry

For the shotgun proteomics analysis, six cell pellets were subject to differential detergent fractionation as described by McCarthy *et al.* using the detergents digitonin, Tween 20 and SDS [24]. After each detergent application, samples were centrifuged to separate solublized proteins from cellular debris. The insoluble pellet left after treatment was subject to trypsin digestion along with the soluble fractions, but could not be quantified. Fractions were normalized to 20 μg each and trypsin digestion as described by McCarthy *et al.* [24]. Following digestion, each fraction was desalted using a peptide microtrap (Michrom BioResources) according to the manufacturer's instructions. After desalting, each fraction was further cleaned using a strong cation exchange (SCX) microtrap (Michrom BioResources) to remove any residual detergent, which could interfere with the mass spectrometry. Fractions were dried and resuspended in 10 μL of 2% acetonitrile (ACN), 0.1% formic acid (FA) and transferred to low retention vials in preparation for separation using 1D-LC.

The high performance liquid chromatography (HPLC) equipment used for peptide separation was an Ultimate 3000 (Dionex) operated in 1D-LC mode at a flow rate of 333 nL per min and equipped with a 0.075 mm × 100 mm column packed with Halo C18 material (Michrom BioResources) for reverse phase separation. Each sample was separated using a 4 h gradient from 2% to 50% Acetonitrile with 0.1% formic acid as a proton source. The column was located on the ion source and connected directly to a nanospray emitter to minimize peak broadening. Scan parameters for the LTQ Velos Pro were one MS scan followed by 20 MS/MS scans of the 20 most intense peaks using high energy collisional dissociation as the fragmentation method. Dynamic exclusion was enabled with a mass exclusion time of 3 min and a repeat count of 1 within 30 sec of initial m/z measurement.

### High resolution mass spectrometry

The two cell pellets reserved for high resolution analysis were lysed and digested as described by Wilton *et al*. [25]. Digested peptides were dried, resuspended in 20 mM KH_2_PO_4_, 20% ACN, pH 3 (Buffer A) in 2.5 μL and transferred to low retention vials in preparation for separation using an Ultimate 3000 configured for 2D-LC. Each sample was loaded at 15 μL/min onto an SCX microtrap (Michrom BioResources) for the first dimension of separation, involving SCX steps of Buffer A plus 0, 5, 10, 15, 20, 25, 30, 40, 50, 100, 250, 500, and 1000 mM KCl. For the second dimension of separation, each eluted salt step was desalted with an inline peptide microtrap (Michrom BioResources) with 2% ACN, 0.1% FA at 5 μL/min. Once desalted, the microtrap was switched into line with a fritless nano column (75 μm × ~10 cm) containing C18 media (5 μ, 200 Å Magic, Michrom) manufactured according to Gatlin [26]. Peptides were eluted using a gradient of 2% to 36% ACN, 0.1% FA at 350 nL/min over 60 min and electrospray ionized for analysis using an LTQ FT Ultra mass spectrometer.

A survey scan m/z 350–1750 was acquired in the FT ion cyclotron resonance cell (Resolution = 100,000 at m/z 400, with an accumulation target value of 1,000,000 ions). Up to the 6 most abundant ions (>3,000 counts) with charge states > +2 were sequentially isolated and fragmented within the linear ion trap using collisionally induced dissociation with an activation q = 0.25 and activation time of 30 ms at a target value of 30,000 ions. M/z ratios selected for MS/ MS were dynamically excluded for 30 seconds.

### Peptide identification

Database searches of the mass spectra were performed using both X!tandem [13] and OMSSA [14] algorithms. Spectra were searched against the reference proteome of *Mycoplasma hyopneumoniae* strain 232 (NCBI ftp, Sept. 5, 2012). A randomized version of the protein database was used for calculating false discovery rates. Searches were performed similarly for the LTQ Velos Pro and LTQ FT Ultra data sets, with the only difference being the precursor m/z tolerance being set to 0.4 Da and 10 ppm respectively. Fragment ion tolerance was set to 0.4 Da for all searches. Tryptic cleavage rules were used with up to two missed cleavages. The following potential amino acid modifications were used: 1) carbamidomethylation of Cysteine, 2) single and double oxidation of methionine, 3) phosphorylation of serine, threonine and tyrosine, and 4) water loss from serine and threonine. X!tandem also has an option to automatically test for pyrolidone derivatives of appropriate N-terminal amino acids; this was enabled. Additional file 4: Table S2 contains details on all the parameters used by X!tandem and OMSSA in this analysis. Peptide identifications were accepted as correct if the e-value for each spectrum-sequence match was 0.01 or less. Protein identifications were discarded if only a single peptide sequence was identified; only peptides uniquely identifying each protein were retained.

The *Mycoplasma hyopneumoniae* strain 232 reference genome sequence was downloaded from NCBI (Sept. 5, 2012) to be used as a database for proteogenomic mapping. A 6-frame translation of the genome according to translation code 4 (*Mycoplasmas*) was performed using Perl. Because of software memory constraints, the 6-frame translation was broken into sections 600 amino acids long, each with a 60 amino acid overlap with the previous, to avoid missing peptide identifications which might span sections. Database searches of the mass spectra were performed using both X!tandem [13] and OMSSA [14] algorithms in an identical manner to the protein searches. Peptide identifications were accepted as correct if the e-value for each spectrum-sequence match was 0.01 or less. Spitting the genome translation could cause protein sequences to be split across two or more fasta entries, therefore, all peptides were retained, not only those uniquely identifying each database entry. Entries identified by a single peptide were discarded.

### Gene ontology of DDF fractions

Differential detergent fractionation was designed to separate proteins based on hydrophobicity. In this study, the detergents digitonin, Tween 20 and SDS were used in the order listed, of increasing strength, to prepare cells for low resolution analysis using the LTQ Velos Pro. Cells prepared for analysis using the LTQ FT Ultra were lysed and digested with no prefractionation. Identified proteins were organized by 1) DDF fraction, 2) all fractions combined and 3) non-DDF. GORetriever, an online tool available on AgBase ( http://agbase.msstate.edu/) [26], was used to collect GO cellular component terms for the three catagories as well as all 691 known *M. hyopneumoniae* proteins.

### Proteogenomic mapping

Proteogenomic mapping was implemented using Perl to match identified peptide sequences to the NCBI reference genome for *Mycoplasma hyopneumoniae* stain 232 (NCBI ftp, Sept. 5, 2012). All identified peptide sequences were string matched to the 6-frame translations. The frame, direction and coordinates of each match were compared to the current annotation general feature format (GFF) file accompanying the genome download and subsequently sorted into preliminary categories. Matches in the same frame and within the boundaries of annotated ORFs were categorized as "annotated ORF". "ORF extensions" were defined by matches in frame with and overlapping the start coordinates of an ORF. "Intergenic" matches fell outside ORF coordinates. "Out-of-frame" matches were defined as any match within or overlapping an ORF, but in a different frame on the same strand. "Opposite strand" matches were also defined as any match within or overlapping an ORF, but on the complement strand. Once all matches were categorized, a GFF file was created allowing these to be viewed along side the current annotations in a genome browser for manual evaluation if necessary. "Annotated ORF" matches were discarded from further analysis since no new information is derived from these. All other types, "ORF extension", "Intergenic", "Out-of-frame" and "Opposite strand" matches were compiled into physically associated groups defined here as "mappings". To create mappings, each frame was scanned and matches between stop codons grouped together. The closest start and stop codons containing each group of matches were recorded; if no start was found, the start of the first match was used. When intergenic matches were grouped with any other type, the other type took precedence as the mappings final category. Any mapping with only a single peptide was discarded.

Prodigal, a prokaryote gene finding software, was used to analyze the *M. hyopneumoniae* genomic sequence to detect ribosomal binding sites and start codons [27]. These predictions were compared to the start codons predicted through the proteogenomic mapping process using Perl.

## Availability of supporting data

Mass spectra and protein identifications have been deposited to the ProteomeXchange Consortium ( http://proteomecentral.proteomexchange.org) via the PRIDE partner repository [28] with the dataset identifier PXD000118 and DOI 10.6019/PXD000118. Results from protein and genomic translation searches, are included as supporting information in tab-delimited format.

## Electronic supplementary material

Additional file 1: Table S1: Mycoplasma hyopneumoniae strain 232 proteins with mass spectrometry verification status and coverage metrics. (XLSX 40 KB)

Additional file 2: **Proteome search results.** (TXT 6 MB)

Additional file 3: **Genome search results.** (TXT 6 MB)

Additional file 4: Table S2: Search parameters. (XLSX 8 KB)

## Abbreviations

ORF: Open reading frame
GFF: General feature format
1D: One dimensional
2D: Two dimensional
LC: Liquid chromatography
NSI: Nanospray ionization
MS/MS: Precursor and fragment mass spectrometry
Velos: LTQ Velos Pro mass spectrometer
FT: LTQ FT (Fourier Transform) Ultra mass spectrometer
SCX: Strong cation exchange
ACN: Acetonitrile
FA: Formic acid
HPLC: High performance liquid chromatography.
